# Analyses clinico-biologiques et suivis électrocardiographiques de 104 cas de paludisme à *plasmodium falciparum* traités au Camp Kosseï de N'Djamena (Tchad)

**DOI:** 10.48327/mtsi.2021.163

**Published:** 2021-11-25

**Authors:** Dorian CELLARIER, Frédéric PONS, Marion REBARDY, Jessica PAEZ, Sarah ROMMEL, Carmelo MOSCATO, Natacha GANTOIS, Eléna LABOURDERE, Rhiannon HOWE, Gilles CELLARIER

**Affiliations:** 1 10^e^ Centre médicale des armées (CMA) de Marseille, France; 2 Hôpital d'instruction des armées (HIA) Sainte Anne de Toulon, France; 3 7^e^ CMA d'Avord, France; 4 11^e^ CMA de Toulouse, France; 5 8^e^ CMA de Clermont-Ferrand, France; 6 HIA Bégin de Saint Mandé, France; 7 12^e^ CMA de Bordeaux-Mérignac, France; 8 15^e^ CMA de Rennes, France

**Keywords:** Paludisme, *Plasmodium falciparum*, Combinaison thérapeutique à base d'artémisinine, Pipéraquine-arténimol, Pipéraquine-dihydroartémisine-triméthoprime, Électrocardiogramme, QTc, Camp Kosseï, N'Djamena, Tchad, Afrique subsaharienne, Malaria, *Plasmodium falciparum*, Artemisinin-based combination therapy, Piperaquine-artenimol, Piperaquine-dihydroartemisine-trimethoprime, Electrocardiogram, QTc, Camp Kosseï, N'Djamena, Tchad, Saharan Africa

## Abstract

**Objectif:**

Le but de cette étude était de déterminer s'il existe, comme admis en pratique clinique, une augmentation significative de l'intervalle QT avec l'utilisation de combinaisons thérapeutiques à base d'artémisinine (CTA) contenant de la pipéraquine. **Méthodologie.** Une étude rétrospective a été réalisée sur les aspects épidémiologiques, cliniques et biologiques, ainsi que sur le retentissement électrocardiographique du traitement par ces CTA chez les patients pris en charge pour paludisme du 31 août au 3 novembre 2017 au pôle de santé unique (PSU) du Camp Kosseï de N'Djamena que soutiennent médicalement les Forces armées françaises engagées au Sahel.

**Résultats:**

Cent-quatre cas patients, traités pour paludisme à ***Plasmodium falciparum*** (28,6 ans [0 - 75 ans], 72 % d'hommes), ont été inclus. Ils étaient tous fébriles (38,4 °C [36,6 - 41,5 °C]), asthéniques, et souvent céphalalgiques et arthromyalgiques (58 %). Il n'y a pas de différence biologique notable avant et après traitement (notamment une kaliémie à 3,81 vs 3,91 mmol/l, p=0,154). Les deux CTA utilisées contenant de la pipéraquine n'ont pas augmenté significativement le QTc (415,8 vs 421,4 ms; p=0,89) et il n'y a pas eu d’événement indésirable notable.

**Discussion:**

L'effectif est majoritairement composé d'hommes tchadiens, ce qui peut biaiser la présentation clinique, car ils sont souvent déjà partiellement immuns. Le suivi médical des militaires français en opérations extérieures, limite les risques de contre-indications à l'utilisation de ces CTA.

**Conclusion:**

Ces résultats sont en faveur d'une bonne tolérance cardiaque de CTA à base de pipéraquine et fait proposer de ne pas réaliser systématiquement un ECG chez les militaires français en opération extérieure lors de leur utilisation.

## Abstract (en)

**Objective:**

The aim of this study was to assess the commonly accepted potential effects of Artemisinin-based combinaison therapy (ACT) on repolarization and QT.

**Method:**

We realized a retrospective study, evaluating epidemiologic, clinical, biological and electrocardiographic data for patients treated for falciparum malaria, between August 31^st^ and November 3^rd^, 2017 in the Pôle de santé unique on the Camp Kosseï of N'Djamena.

**Results:**

One hundred and four patients were included (28,6 years old [0 - 75 years], 72% male). All had fever (38,4 °C [36,6 - 41,5 °C]), asthenia, and main symptoms were headache and arthromyalgia (58%). No significant difference was noted after treatment concerning biological data (especially kaliemia: 3.81 versus 3.91 mmol/l, p = 0.154). There was no significant increase of QTc (415.8 versus 421.4 ms, p = 0.89) with the two ACT treatment used and no adverse events.

**Discussion:**

Population is essentially composed of Chadian men, often partly immunized, that can modify clinical presentation. French soldiers’ medical follow up in military operations decreases contra-indications of ACT.

**Conclusions:**

These results are in favor of a good cardiac tolerance of ACT with piperaquine and it should be proposed not to realize systematic ECG for the French soldiers in external operation when treated with ACT.

## Introduction

Lors d'un accès palustre simple, le traitement de référence est, depuis 2010 en première intention, une combinaison thérapeutique à base de dérivés d'artémisinine (CTA) [8, 10].

D'après les « mentions légales » du dictionnaire Vidal^**®**^ et les dernières recommandations de la Société de pathologies infectieuses de langue française (SPILF), un CTA à base de pipéraquine (pipéraquine 320 mg + dihydroartémisinine 40 mg: Eurartésim^**®**^) peut être responsable d'un allongement du QT, mais sans conséquence clinique (notamment en termes d'arythmie: torsade de pointe par exemple) dans les études réalisées. En outre, le plan de gestion des risques du laboratoire préconise des modalités de surveillance de la toxicité cardiaque « lorsque cela est cliniquement pertinent ». La SPILF précise même que le traitement peut être envisagé en ambulatoire si les conditions cliniques, biologiques et sociales sont réunies [10]. Les contre-indications des CTA comprennent bien entendu les antécédents de QT long et d'arythmies cardiaques.

Pour rappel, les principales causes d'allongement du QT sont: les médicaments (anti-arythmiques de classe Ia et III, certains psychotropes, certains anti-infectieux, les antipaludéens avec principalement la chloroquine, etc.), les troubles électrolytiques (hypokaliémie et hypocalcémie), les anomalies congénitales avec notamment le syndrome du QT long congénital, et de nombreuses autres causes diverses (bloc auriculo-ventriculaire de 3^e^ degré, insuffisance coronarienne, hypothermie, hypothyroïdie, prise de cocaïne au long cours…).

La Société française de médecine des armées (SFMA) conseille l'utilisation en première intention de la CTA à base de pipéraquine 320 mg, dihydroartémisinine 40 mg (Eurartesim^**®**^) et de réaliser systématiquement un électrocardiogramme (ECG) en pré-, per- et post-thérapeutique en raison des risques de trouble du rythme cardiaque liés à l'allongement potentiel du QT (QTc) [6]. En pratique, sur les théâtres d'opérations en zone endémiques ces recommandations limitent l'utilisation de cette CTA par les médecins militaires français, qui lui préfèrent l'association atovaquone-proguanil (Malarone^**®**^), d'utilisation moins contraignante, recommandée pourtant par la SFMA seulement en deuxième intention [9].

Pourtant un allongement du QT à risque avec une CTA parait rare et ce d'autant plus qu'aucun retentissement à type de troubles du rythme n'a été retrouvé dans les différents articles de notre connaissance [1,2,4,5,7,11].

Au Tchad, le paludisme (avec une prédominance durant la saison des pluies) reste encore et toujours une des premières causes de morbi-mortalité avec la malnutrition infantile et les accidents de la route entre autres. Il demeure la maladie qui tue le plus, devant notamment la rougeole [3]. Les médecins militaires du PSU du Camp Kosseï de N'Djamena prennent en charge de nombreux cas de paludisme en saison des pluies. C'est dans ce contexte que nous avons analysé de manière rétrospective les aspects cliniques, biologiques, évolutifs et le retentissement électrocardiographique des CTA comprenant de la pipéraquine, chez 117 patients (104 inclus), traités pour paludisme à *P. falciparum* au PSU de N'Djamena entre le 31 août et le 3 novembre 2017.

## Méthodologie et population étudiée

Nous avons réalisé une étude rétrospective monocentrique concernant tous les patients pris en charge au PSU de N'Djamena entre le 31 août et le 3 novembre 2017 pour paludisme à *Plasmodium falciparum.*

Nous avons étudié la population ayant reçu effectivement une CTA contenant de la pipéraquine et ayant eu un suivi clinique, biologique (complet pour la majorité d'entre eux) et exclusivement celle ayant eu un ECG avant traitement et un ECG après traitement.

Le diagnostic, évoqué sur des signes cliniques et/ou fièvre compatibles, était confirmé par test de dépistage rapide (TDR) de type « PaluTop^**®**^ ». Il était systématiquement confirmé par frottis. En cas de forte suspicion clinique, si ce frottis se révélait négatif, une recherche sur « goutte épaisse » (GE) était effectuée par un technicien de laboratoire militaire français ayant une grande expérience du paludisme de par ses déploiements en OPEX (pas de biologiste disponible). Ni le « Quantitative Buffy Coat » (QBC Malaria^**®**^), ni la recherche de sérologies de paludisme n’étaient disponibles.

L'interrogatoire recherchait les antécédents de paludisme, la durée de la symptomatologie, la prise d'une chimioprophylaxie. L'examen clinique comprenait le recueil de la température, la mesure du poids, la prise de la tension artérielle, un examen digestif, neurologique, pulmonaire et cardiovasculaire. Chez tous les patients était prescrit un bilan biologique avant traitement (le jour même) et après la dernière prise d'antipaludéen. Il comportait: une biologie standard (avec notamment NFS, plaquettes, ionogramme sanguin, fonctions rénales et hépatiques, CRP), et un frottis à la recherche de trophozoites. Il n’était pas possible techniquement de réaliser d'hémoculture.

Un ECG 12 dérivations était réalisé chez tous les patients, avec l'appareil disponible sur place, un Schiller^®^ AT-102 (mise en service 2010 avec contrôle technique annuel), avant la première prise d'antipaludéen, et le dernier jour du traitement après la dernière prise. Cet appareil mesure le QTc selon la formule de Bazett. L'ECG per-thérapeutique n'a pas été réalisé du fait, d'une part des contraintes opérationnelles et, d'autre part des déplacements souvent difficiles des consultants autochtones. L'interprétation a été réalisée par un cardiologue rythmologue pour tous les tracés.

Le QTc est considéré comme prolongé lorsqu'il est supérieur à 450 ms chez l'homme et 470 ms chez la femme. Le risque de torsades de pointe est considéré comme étant significatif lorsque le QTc est prolongé au-delà de 500 ms [12].

Tous les patients pris en charge pour paludisme à *P. falciparum* étaient traités en 1ère intention par pipéraquine 320 mg + arténimol 40 mg (Eurartésim^**®**^) ou pipéraquine 320 mg + dihydroartémisine 32 mg + triméthoprime 90 mg (Artécom^**®**^) selon les disponibilités de la pharmacie.

En cas d'intolérance digestive et/ou de paludisme grave, ou de vomissements persistants 30 mn après administration d'antiémétiques, la quinine IV était utilisée.

L'atovaquone 62,5 mg + proguanil 25 mg (Malarone^**®**^) était prescrite chez l'enfant de moins de 7 kg sans signe de gravité.

L'Eurartésim^**®**^ et l'Artécom^**®**^, le deuxième prescrit lors de rupture de stock du premier au bénéfice de la population locale uniquement, étaient administrés selon une posologie adaptée au poids, en une prise orale unique quotidienne à jeun, 3 jours consécutifs à heure fixe. Un antiémétique (métoclopramide ou ondansétron) était proposé si nécessaire avant ingestion du traitement, et le patient était surveillé pendant une heure après la prise de l'antipaludéen pour s'assurer de l'absence de vomissement; le cas échéant, une nouvelle demi-dose de CTA était prescrite si des vomissements survenaient entre 30 mn et 1 h ou un traitement par quinine IV était réalisé en cas de vomissements dans la demi-heure suivant la prise.

Un traitement antipyrétique et antalgique par paracétamol était systématiquement associé, ainsi qu'un traitement symptomatique en cas de troubles digestifs, avec si nécessaire une réhydratation intraveineuse.

L'ensemble de la prise en charge (médicale et paramédicale) des patients était entièrement réalisée par le PSU et donc les Forces armées françaises.

Les analyses statistiques ont été effectuées par le test « t » de Student bilatéral pairé. Une valeur de « p » < 0,05 était retenue comme statistiquement significative.

Tous les patients (ou parents de mineur) avaient donné leur accord oral pour une analyse anonymisée a posteriori des données de leur dossier clinique.

Nous avons eu l'accord de la Commission de validation des études cliniques de l'Hôpital d'Instruction des Armées (HIA) Sainte Anne (IRB 00011873) pour réaliser ce travail.

Les 2 CTA à base de pipéraquine employées ont une autorisation de mise sur le marché (AMM) au Tchad.

## Résultats

Cent dix-sept patients ont été suivis: 5 Français (dont 2 militaires) et 112 Tchadiens dont 3 militaires tchadiens et 52 personnels civils tchadiens travaillant dans le camp (PCT). Parmi eux, 113 ont bénéficié d'une CTA à base de pipéraquine, 2 ont reçu de la quinine IV et 2 autres l'association atovaquone-proguanil; 104 ont pu avoir un suivi clinique et ECG complet; 8 d'entre eux, exclusivement des Tchadiens non-PCT, ne se sont pas présentés à la consultation de suivi, et l'ECG post-traitement n'a pas pu être effectué sur un enfant agité.

Au total, 104 personnes dont 75 hommes (soit 72 %) et 29 femmes, avec un âge moyen de 28,6 ans (allant de 9 mois à 75 ans, et comprenant 32 mineurs), ont été retenues. Parmi elles, on compte les 5 Français et 99 Tchadiens. On note 74 % de patients ayant eu au moins un antécédent de paludisme (uniquement dans la population tchadienne). Le poids était en moyenne de 59,7 kg.

Les symptômes les plus fréquents étaient une asthénie, des céphalées et des arthromyalgies. La durée moyenne des signes fonctionnels était de 4,6 jours. La fièvre était un signe constant avec une température moyenne de 38,4 °C (allant de 36,6 °C à 41,5 °C: 28 personnes étaient apyrétiques, car avaient déjà pris de leur propre chef un antipyrétique avant leur prise en charge sur le camp). L'asthénie était constatée cliniquement dans tous les cas et importante dans 50 % des cas. Les patients avaient ensuite par ordre de fréquences: des céphalées et arthromyalgies (58 %), des nausées et/ou vomissements (46 %), des diarrhées et/ou douleurs abdominales (31 %) et enfin une toux (11 %).

On relève dans cette étude 100 % d'infections à *P. falciparum,* parmi lesquelles 4 co-infections à P. vivax. La parasitémie était en moyenne de 0,27 % (allant de < 0,001 % à 5,4 %). Aucune différence significative concernant les analyses biologiques avant et après traitement n'a été constatée. C'est notamment vrai pour la kaliémie: 3,81 mmol/l avant traitement versus 3,91 mmol/l après traitement (p=0,154). La CRP était très variable, ceci pouvant être expliqué par la présence de co-infections (comme la typhoïde par exemple), mais en l'absence d'hémocultures disponibles, cela n'a pu être confirmé. Concernant le taux de plaquettes, il était en moyenne de 160,6 G/l avant traitement (avec 48 patients sous le seuil des 150 G/l), contre 178,2 G/l après le traitement (dont 36 patients sous le seuil des 150 G/l).

Le test PaluTop^**®**^ était positif dans 98 % des cas. Il était systématiquement confirmé par frottis. Deux faux négatifs ont été corrigés par la GE.

Enfin, 5 patients ont reçu un traitement par CTA malgré un paludisme « grave », parmi lesquels 4 étaient considérés comme tel sur des critères biologiques uniquement (notamment la parasitémie).

**Figure 1:**
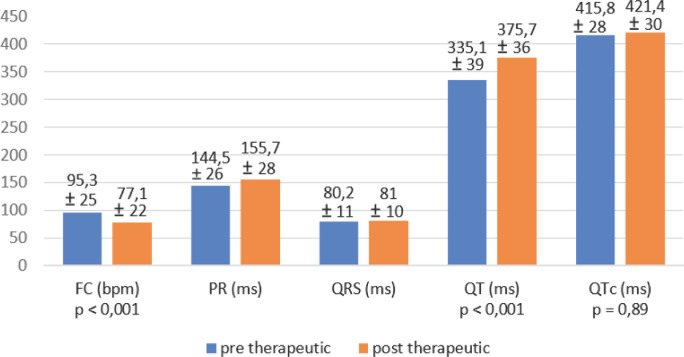
Données électrocardiographiques Echocardiographic data

Deux cent trente-quatre ECG ont été réalisés, dont 208 pour l'effectif retenu. Tous étaient en rythme sinusal (avant et après traitement). Sept patients avaient un intervalle QTc initial qualifié de long, et ont tout de même reçu une CTA en raison d'un QT en valeur absolu court. La fréquence cardiaque était globalement diminuée après le traitement, passant en moyenne de 95 bpm à 77 bpm (p < 0,001), et la largeur du QRS restait inchangée. Le QT était augmenté de façon significative passant de 335 ms en moyenne à 375 ms (p < 0,001), ce qui n'avait pas de retentissement clinique. En revanche le QTc n’était pas augmenté de façon significative, passant de 415,8 ms avant traitement à 421,4 ms après le traitement complet par CTA (p = 0,89) (Fig. 1). Après traitement, 9 personnes avaient un QTc allongé, dont une seule avec un QTc supérieur à 500 ms (sans complication rythmique cardiaque); toutes avaient en revanche un QT non corrigé dans la norme.

Le traitement administré a été bien toléré et tous les patients ont guéri d'un point de vue clinique. Il n'y a pas eu d'effet indésirable grave, notamment sur le rythme (pas de torsades de pointe). Il n'y a pas eu de cas recensé de résistance du *P. falciparum* à aux CTA employés.

## Discussion

La présentation clinique et biologique de cette série est similaire aux notions connues du paludisme à *P. falciparum* [8].

L'effectif est principalement composé de Tchadiens, et majoritairement d'hommes du fait que les employés locaux de la base sont majoritairement des hommes. Cette majorité de Tchadiens peut biaiser la présentation clinique, car ils sont souvent déjà partiellement immuns, ce qui peut diminuer la gravité de la présentation clinique et biologique, et fausser les tests diagnostiques.

Dans ce travail, on ne compte seulement que 2 faux négatifs du test de dépistage rapide Palutop^**®**^ chez 2 patients qui avaient par ailleurs déjà débuté un traitement en ville, rendant la sensibilité du test sur cette série à 98 %.

En revanche, la faible prévalence de paludisme dans la population expatriée présente dans le camp, met en avant l'efficacité de la chimioprophylaxie par doxycycline associée au reste des mesures de prévention anti-vectorielle. Sur presque 1 000 français soutenus par le PSU et prenant la doxycycline, seulement 5 ont fait un accès palustre. Tous les 5 ont reconnu une mauvaise observance ou une inobservance de la chimioprophylaxie. A contrario, le PSU a diagnostiqué un paludisme à *P. falciparum* chez 49 PCT sur les 250 présents sur base, pourcentage élevé malgré leur statut le plus souvent immun.

Les résultats de ce travail chez des patients traités pour accès palustre à *P. falciparum,* ne montrent pas d'allongement significatif du QTc après la fin du traitement par CTA à base de pipéraquine (415,8 ms avant traitement versus 421,4 ms après le traitement; p = 0,89). Il n'y a pas eu non plus d'effet indésirable à type de torsades de pointe. Le QT non corrigé était quant à lui significativement plus court avant le traitement qu'après (335 ms versus 375 ms; p < 0,001). Ceci est expliqué par la tachycardie initiale (due à la fièvre, la déshydratation, l'inflammation et aux arthromyalgies notamment), ce qui diminue la durée de la valeur absolue du QT. Il faut également rappeler que cette fréquence cardiaque initialement élevée avant traitement, exagère le QTc. En effet, la formule de Bazett (QTc=QTm/√RR’) qui est utilisée pour calculer le QTc est valable pour une fréquence cardiaque de 60 bpm. Contrairement à celle de Framingham, elle sous-estime le QTc pour des fréquences inférieures et au contraire le surestime pour des fréquences plus hautes. Il faut donc savoir être critique envers un QTc initialement qualifié de long, lorsque la fréquence cardiaque est élevée, avant de ne pas introduire le traitement recommandé. De plus, la valeur absolue du QT (étant normal) dans ce cas associé à la tachycardie rend le risque de torsades de pointe très faible.

Les militaires français en opérations extérieures (OPEX) ont tous avec eux un livret médical réduit (LMR) comprenant un un ECG de référence (qui a au minimum moins de 4 ans) et leurs antécédents. Un syndrome du QT long et/ou une arythmie cardiaque rendent inapte un militaire à partir en OPEX. L'intérêt de rester sur des anciennes recommandations lors de la prise en charge d'un paludisme simple chez les militaires français en OPEX, limitant en pratique l'utilisation du traitement de première intention, peut donc être discuté, voire remis en cause. Il existe actuellement au sein du Service de santé des armées des travaux pour optimiser la prise en charge thérapeutique du paludisme.

Des travaux incluant de plus grands effectifs sur le terrain, et notamment chez les forces armées en opération extérieure atteint de paludisme non grave à *P. falciparum,* permettrait certainement de conforter l'absence de risque cardiaque lors d'un traitement par CTA dans cette population. En attendant, il ne parait pas déraisonnable, sur les bases de ce travail préliminaire et des pratiques médicales en milieu civil, de proposer de se passer de la réalisation systématique d'ECG, lors de prescription des CTA contenant de la pipéraquine pour un accès palustre non compliqué chez nos militaires hors métropole. L'utilisation du traitement de première intention recommandé pourrait alors être favorisée dans des postes isolés sans ECG disponible.

Cette étude présente plusieurs limites. Elle est premièrement rétrospective, avec un effectif relativement limité.

Le contexte opérationnel ne permettait pas de suivre exactement les recommandations dans tous les cas. Il manque notamment le 2^e^ ECG de contrôle. L'absence de recherche de co-infection bactérienne est également un biais, tant sur le plan clinique que biologique pouvant peut-être expliquer la fréquence des formes digestives.

## Conclusion

Les résultats de cette étude sont en faveur de l'efficacité et de la bonne tolérance clinique, biologique et rythmique des CTA à base de pipéraquine, n'allongeant pas dans notre étude le QTc.

Ils peuvent ainsi faire proposer de ne pas réaliser systématiquement d'ECG lors d'utilisation des combinaisons thérapeutiques à base de dérivés d'artémisinine chez les militaires français opérant en zone d'endémie palustre sans risque de QT long.

Ce travail, à l'origine d'une thèse de médecine, a contribué à la modification de l'utilisation de l'Eurartesim^**®**^ dans les armées françaises lors du traitement d'un accès palustre, avec l'arrêt de la surveillance ECG systématique (instruction actée en cours d’écriture).

## Remerciements

Les auteurs remercient les personnes suivantes pour leur participation à l’étude:

- ROMANAT Pierre-Emmanuel
- DEMOURES Thomas
- SCHLIENGER Guislain
- LE GOFF Pierre-André
- MADEC Samuel
- PY Emmanuel
- GRANGER-VEYRON Nicolas
- BAUDOIN François

## Liens d'intérêts

Les auteurs déclarent ne pas avoir de liens d'intérêt.

## Contribution des auteurs

- CELLARIER Dorian: analyse des données, interprétation des résultats, rédaction
- PONS Frédéric: supervision de l’étude, analyse des données, validation du protocole
- REBARDY Marion: inclusion des sujets, suivi clinique, surveillance thérapeutique
- PAEZ Jessica: inclusion des sujets, suivi clinique, surveillance thérapeutique
- ROMMEL Sarah: inclusion des sujets, suivi clinique, surveillance thérapeutique
- MOSCATO Carmelo: exécution des tests de laboratoire
- GANTOIS Natacha: inclusion des sujets, suivi clinique, surveillance thérapeutique
- LABOURDERE Eléna: inclusion des sujets, suivi clinique, surveillance thérapeutique
- HOWE Rhiannon: inclusion des sujets, suivi clinique, surveillance thérapeutique
- CELLARIER Gilles: conception de l’étude, inclusion des sujets, suivi clinique, surveillance thérapeutique, validation des données, interprétation des résultats, supervision de l’étude, co-rédaction
